# Curative effect of acupuncture combined with Chinese medicine on cancer pain: Systematic review and meta-analysis

**DOI:** 10.1097/MD.0000000000048463

**Published:** 2026-05-08

**Authors:** Guocheng Xue, Siqi Zhang, Afeng Miao, Yubo Teng, Hao Ming, Miao Zhang

**Affiliations:** a Heilongjiang University of Chinese Medicine, Harbin, Heilongjiang, China; b Acupuncture and Moxibustion Consulting Room No. 10, The Second Affiliated Hospital of Heilongjiang University of Chinese Medicine, Harbin, Heilongjiang, China.

**Keywords:** acupuncture combined with Chinese medicine, cancer pain, integrative medicine of traditional Chinese medicine

## Abstract

**Background:**

Cancer and cancer pain caused by treatment have brought great challenges. With the increasing number of papers on the treatment of cancer pain with acupuncture combined with Chinese medicine, this paper aims to evaluate the evidence of the association of acupuncture combined with Chinese medicine with reduction in cancer pain.

**Method:**

Four English databases and 4 Chinese databases were systematically searched from the establishment of the database to September 18, 2024. Randomized clinical trials that compared acupuncture combined with Chinese medicine with acupuncture alone, analgesic therapy, or other usual care for cancer pain were included. Data were screened and extracted independently using predesigned forms. The quality of randomized controlled trials (RCTs) was appraised with the Cochrane Collaboration risk of bias tool with Rvman 5.4 software. According to the different types of data,count data used risk ratioas the effect sizes, and Measurement data used Mean difference (MD)as the effect sizes and standardized MD.

**Results:**

Ten RCTs (comprising 884 participants) were incorporated into the systematic review, with data from all ten trials included in the meta-analysis. Findings from 7 of the trials indicated that the combination of acupuncture and Chinese medicine, when compared to other forms of care, led to a decrease in pain intensity. A good analgesic efficacy was seen by using the acupuncture combined with Chinese medicine in 6 RCTs (MD, 1.22 points; 95% confidence interval [CI], 1.12 to 1.32;*Ⅰ*^2^ = 13%). Two RCTs showed that acupuncture combined with Chinese medicine and tertiary analgesic therapy could shorten the onset time of analgesics(MD, −14.82 points;95% CI, −16.80 to −12.82;*Ⅰ*^2^ = 0%); and 3 RCTs increased the duration of analgesia(MD, 2.40 points;95% CI, 2.01 to 3.82;*Ⅰ*^2^ = 95%). An improvement in the quality of life of the cancer patients was seen when acupuncture combined with Chinese medicine was used in 5 RCTs (MD, 4.68 points;95% CI, 3.69 to 5.66;*Ⅰ*^2^ = 0%).

**Conclusion:**

The systematic review and meta-analysis conducted that a significant reduction in cancer pain was observed in patients who received acupuncture combined with Chinese medicine, compared to those who received other treatment.

## 1. Introduction

Cancer pain is classified as a nociceptive pain, caused by ongoing tissue damage; or a type of neuropathic pain, resulting from tumor masses compressing peripheral nerves and injury nerves system of radiotherapy and chemotherapy.^[1,2]^ Cancer pain management is a critical aspect of oncology care, as it significantly impacts patients quality of life and overall well-being. The prevailing approaches to cancer pain management often involve a multifaceted strategy that incorporates pharmacological interventions, non-pharmacological therapies.^[3–8]^

Acupuncture is recommended for a wide range of clinical conditions. In particular, it has beneficial effects on pain control, and more importantly is safe to use. The benefits of acupuncture include rapid onset of analgesic effect, long sustained remission, ease of application, no risk of drug dependence or addiction, and by and large, the absence of serious side effects. Nowadays, acupuncture is utilized in all phases of cancer pain management.^[9,10]^ Chinese medicine therapy not only alleviates the symptoms (e.g., fatigue, chronic pain, anorexia/cachexia, and insomnia) of patients with cancer and improves their quality of life but also diminishes adverse reactions and complications caused by chemotherapy, radiotherapy, or targeted therapy. Acupuncture combined with Chinese medicine therapy not only reduces cancer pain, but also reduce adverse reactions.^[11,12]^ There has been no rigorous systematic evaluation of the effect of acupuncture combined with Chinese medicine on cancer pain, so we conducted a systematic review and meta-analysis of the available evidence to guide clinical practice.

Given the increasing quantity of randomized controlled trials (RCTs) examining the effectiveness of acupuncture in conjunction with Chinese medicine for alleviating cancer-related pain, a systematic review and meta-analysis were carried out to assess the existing evidence and provide guidance for clinical decision-making. The primary research inquiries were: does the utilization of acupuncture combined with Chinese medicine lead to a decrease in cancer pain compared to solely utilizing acupuncture or alternative forms of care control? does the integration of acupuncture combined with Chinese medicine result in a reduction in the consumption of analgesics among cancer patients?

## 2. Method

Registered in PROSPERO (International prospective register of systematic reviews) under the identifier CRD42024591190, this meta-analysis and systematic review evaluated RCTs investigating the efficacy of acupuncture combined with Chinese medicine for pain management in cancer patients. The Cochrane Collaboration’s risk of bias tool was utilized to evaluate the quality of the trials, while the Grading of Recommendations Assessment, Development, and Evaluation approach was applied to assess the overall evidence and certainty.

### 2.1. Search strategy and study selection

The following electronic databases were searched from inception to September 18, 2024: 4 English-language databases (PubMed, Embase, Cochrane library and CINAHL)and 4 Chinese-language databases(China Biology Medicine Disc, VIP Chinese Science and Technology Journal Database, China National Knowledge Infrastructure, Wanfang Med). The search strategy was based on the PICOS (Population\Intervention\Comparison\Outcome\Study design) principle and consisted of 4 components: clinical condition (cancer pain*, cancer-related pain*, cancer-associated pain*, neoplasm-related pain*, neoplasm-associated pain*, tumor-related pain*, tumor-associated pain*, oncological pain* and oncology pain*), intervention (acupuncture, traditional Chinese medicine (TCM), acupuncture medication combined, acupuncture combined with TCM, auricular acupuncture, and electroacupuncture), outcome (pain, ache, and analgesia), and study type (RCT, random*, controlled clinical trial). We analyzed previous systematic reviews to find more trials. G.C.X. and A.F.M. independently reviewed search results to determine study eligibility by titles, abstracts, or full text. If we disagree, a third person (M.Z.) decides the eligibility of the article for the study. Included were English or Chinese language articles that were RCTs examining the relationship between acupuncture/acupressure and cancer pain, encompassing various trial designs such as blinding, crossover, and pragmatic trials. The focus was on pain related to cancer progression or treatment-induced chronic pain. Studies on acute pain management post-surgery were not considered. The eligible intervention was acupuncture combined with Chinese medicine, and acupuncture included manual acupuncture and acupressure, electroacupuncture, auricular acupuncture, or a combination of these techniques regardless of acupuncture technique and stimulation method, and Chinese medicine included external application, fumigation, or decoction. The comparison was simple acupuncture therapy, conventional pain management and other active pain treatments (such as yoga, exercise, etc). Excluded were studies comparing herbal preparations through acupuncture point injection with a single Chinese medicine therapy like herbal medicine or massage. Pain intensity was the chosen outcome due to its significant role in evaluating^[1]^ and managing cancer pain. Outcome measures comprised improvement rates, numerical rating scale, visual analog scale, and other validated tools for pain intensity assessment.

### 2.2. Data extraction and quality assessment

Data extraction was conducted independently by 2 researchers (G.C.X. and Y.B.T.) using predefined forms. The extraction process involved capturing clinical features such as participant demographics, intervention specifics, and outcome measurements. Additionally, details regarding treatments, methodological attributes, and outcome results were systematically extracted for each study. Quality assessment of the included studies was independently performed by 2 researchers (G.C.X. and A.F.M.), with any discrepancies resolved through discussion and consensus with a third reviewer (M.Z.). Each RCT was assigned a low, high, or unclear risk of bias through Rvman 5.4 software in 6 specific areas (sequence generation, allocation concealment, blinding of participants and outcome assessments, incomplete outcome data, selective outcome reporting, and other potential threats) using information identified from published articles and supplementary materials, and study authors were contacted as needed.

### 2.3. Data synthesis plan

Meta-analysis of RCTs with available data was performed by calculating effect sizes. According to the different types of data,count data used risk ratio as the effect sizes, and measurement data used mean difference (MD) as the effect sizes and standardized MD was used when the measurement unit and method were different. All statistical analyses were conducted using R v5.4 and 95% confidence interval (CI). Heterogeneity among trials was identified by χ^2^ the test and reported as*Ⅰ*^2^. If*Ⅰ*^2^ ≤ 50%, the heterogeneity was considered to be small, and the fixed effect model was used for analysis. Otherwise, the heterogeneity was considered to be high, and the random effects model was used for analysis, and the source of heterogeneity was further analyzed. Subgroup sensitivity analyses were conducted to explore potential sources of heterogeneity. When possible and appropriate, planned subgroup analyses included the source of pain (cancerous organ or specific treatment), severity of pain (mild, moderate, or severe), and type of treatment (manual acupuncture, electroacupuncture, or auricular acupuncture). Publication bias was assessed by funnel plots for asymmetry when at least 10 trials were included.

## 3. Result

### 3.1. Included results and characteristics of included articles

**Figure 1.**
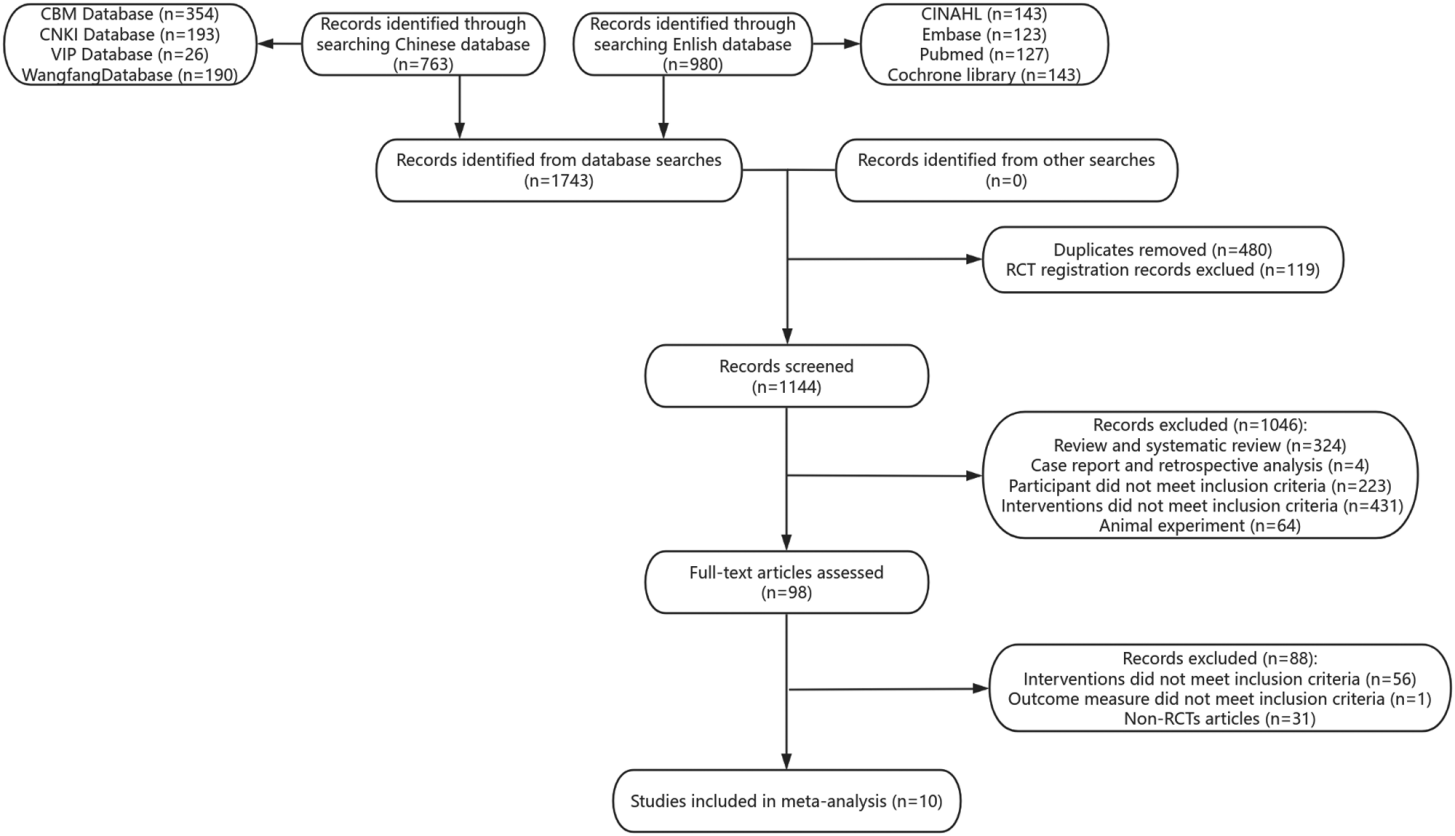
Flow Chart of All Enrolled RCTs. n = number of records, RCT = randomized control trial.

1743 records were identified through database and other searches. After removing 480 duplicate publications and excluding 119 articles for RCT registration records, 1046 studies were excluded following abstract review. This left 98 studies for detailed eligibility review. Finally, 10 articles^[13–22]^ were included in the systematic review or qualitative synthesis(Fig. 1).

Regarding the sources of article, all 10 articles were sourced from China. A total of 884 participants were included in 10 articles for statistical analysis, including 395 participants in the intervention group and 487 participants in the control group. Regarding participant diagnoses, 5 articles reported multiple cancers, 1 article reported bone metastatic cancer, and 4 articles reported a single type of cancer. Regarding intervention measures, 5 articles compared the efficacy of acupuncture combined with TCM against a 3-stage analgesia protocol, 2 articles compared the analgesic effect of oxycodone hydrochloride sustained release tablets, 2 articles compared acupuncture combined with TCM to either acupuncture or TCM alone, and 1 article compared with anther drug.Regarding acupuncture manipulation, 3 articles were electroacupuncture therapy, 7 articles were manual acupuncture and the specific acupuncture points are shown in Table 1. Regarding the application of traditional Chinese medicine, 7 articles were external application, 2 articles were decoction, one was for powder preparation, and the specific herbs and methods are shown in Table 2.

**Table 1:** The specific acupoints of the article.

Included literature	Acupuncture point (Code)	Methods of acupuncture
Wenhua Duan 2023^^[13]^^	Ouch point	The pain site was punctured around
Meng Yun, et al 2021^[14]^	Neiguan (PC6); Sanyinjiao (SP6)	Hand needle
Zhilong Wang 2021^[15]^	Ouch point	Hand needle
Xinju Chen, et al 2014^[16]^	Ouch point; and the corresponding Xi-points, yuan-points and back-shu points	Hand needle and electroacupuncture
HE Jing, et al 2024^[17]^	Hegu (LI4); Yanglingquan (GB34); Taichong (LR3); Dazh-ui (DU14); ouch point	Hand needle
Chunpeng Jiang 2017^[18]^	Ouch point; And the corresponding xi-points, yuan-points and back-shu points	Hand needle and electroacupuncture
Yu Wang 2017^[19]^	Ouch point; Zusanli (ST36); and the corresponding back-shu points	Hand needle
Jie Zhou, et al 2017^[20]^	Neiguan (PC6); Kongshu (LU6); Hegu (LI4); Lieque (LU7); Feishu (BL13); Zhongfu (LU1); Zusanli (ST36); Sanyin- jiao (SP6)	Hand needle
Li Yu, et al 2021^[21]^	Guanyuan (RN4); Zusanli (ST36); Taichong (LR3); Weishu (BL21); Geshu (BL17)	Hand needle
Dongbo Cao 2018^[22]^	Eightfold method of the sacred tortoise	Hand needle

**Table 2:** The article on herbs and methods of decoction.

Included literature	Traditional Chinese herbs prescription	Methods of herbs
Wenhua Duan 2023^[13]^	Jianghuang 30 g; Dingxiang 10 g; Ruxiang 10 g; Moyao 10 g; Yanhusuo 15 g; Wugong 3 g; Dannanxing 30 g; Fengfang 10 g; Zaojiaoci 10 g; Dilong 10 g; Shengpuhu-ang 15 g; Xixin 3 g	Make an ointment and apply to the painful area
Meng Yun, et al 2021^[14]^	Huajizhentong ointment (Tiannanxing; Fuzi; Chuan-wu; Maqianzi; Hunagyaozi; Chansu)	Make an ointment and apply to the painful area
Zhilong Wang 2021^[15]^	Dahuang 100 g; Mangxiao 200 g; Bingpian 5 g	Make an ointment and apply to the painful area
Xinju Chen, et al 2014^[16]^	Ruxiang 20 g; Moyao 20 g; Maqianzi 20 g; Shannai 20 g; Yuanhu 30 g; Shexiang 0.1 g	Make an ointment and apply to the painful area
He Jing, et al 2024^[17]^	Jinlingzi 30 g; Yanhusuo 30 g	Grind into powder and take drenched with boiling water
Chunpeng Jiang 2017^[18]^	Ruxiang 20 g; Moyao 20 g; Maqianzi 20 g; Shannai 20 g; Yuanhu 30 g; Shexiang 0.1 g	Make an ointment and apply to the painful area
Yu Wang 2017^[19]^	Gouqi 20 g; Huangqi 20 g; Yanhusuo 20 g; Baihuashe-shecao 30 g; Banzhilian 30 g; Taizishen 15 g; Ezhu 15 g; Baishao 15 g; Shenqu 15 g; Chuanlianzi 15 g; Shanzha 15 g; Fuling 15 g; Buguzhi 15 g; Maiya 15 g; Muxiang 10 g; Quanxie 10 g; Danggui 10 g	Decoct with water
Jie Zhou, et al 2017^[20]^	Wenhuagao ointment (Huangbai; Wubeizi)	Make an ointment and apply to the painful area
Li Yu, et al 2021^[21]^	Huangqi 50 g; Danggui 10 g; Chuanxiong 10 g; Baishao 15 g; Shijianchuan 15 g; Tengligen 15 g; Ezhu 15 g; Yin- yanghuo 15 g; Shemei 10 g	Grind into powder and take drenched with boiling water
Dongbo Cao 2018^[22]^	Gancao 100 g; Banbianlian 80 g; Banzhilian 80 g; Danshen 60 g; Baihuasheshecao 60 g; Baishao 60 g; Quanxie 50 g; Sanleng 50 g; Ezhu 50 g; Gansui 50 g; Wugong 30 g; Shuduchong 30 g; Bingpian 10 g; Maqianzi 5 g; Banmao 5 g	Make an ointment and apply to the painful area

### 3.2. Quality of Evidence

**Figure 2.**
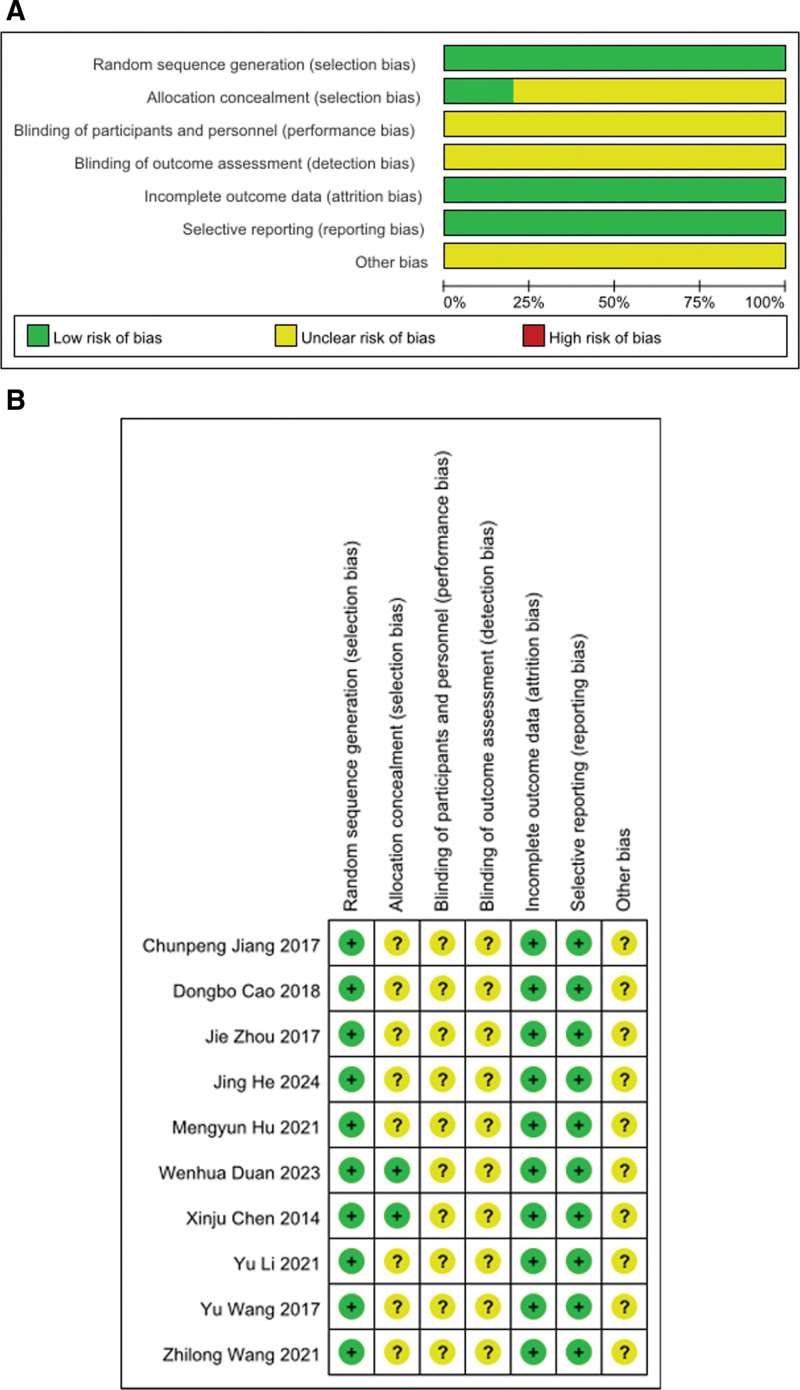
Risk of bias graph and risk of bias summary.

10 articles were medium quality, as blinding method in these articles did not mention,and did not address other risks, but in complete outcome data judged to have a low risk of bias. In terms of randomized methods, these articles were judged to have a low risk of bias, 4 articles used random number table, 4 articles used block randomization, 1 article used Sealed envelope and 1 article used Computer random. In these articles of allocation concealment, 2 articles used random card assignment, but the other articles did not mention random assignment (Fig. 2A and Fig. 2B).

### 3.3. Outcome of acupuncture combined with Chinese medicine and other therapies

**Figure 3.**
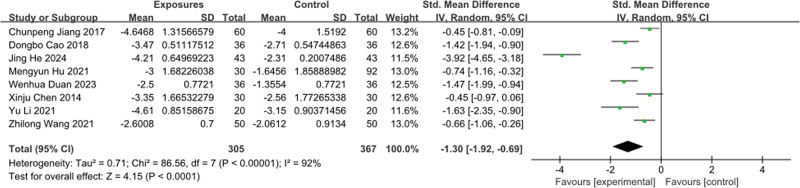
Pain intensity score forest map. CI = confidence interval, SD = standard deviation.

With regard to pain intensity score (Fig. 3), 8 studies^[13–18,21,22]^ used numerical descriptor scale score and 1 study used visual analogue scale score, so standardized mean difference served as the primary efficacy statistic. The combined results from 8 studies revealed a connection between pain alleviation and the integration of acupuncture with Chinese medicine, as opposed to other treatments, exhibiting significant heterogeneity. (MD, −1.30 points; 95% CI, −1.92–−0.69;*Ⅰ*^2^ = 92%).

**Figure 4.**
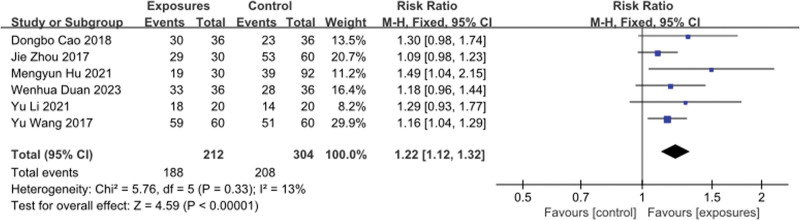
Pain relief rate forest map. CI = confidence interval, M-H = Metropolis-Hasting algorithm.

With regard to pain relief (Fig. 4), 6 studies^[13,14,19–22]^ both used the number of pain relief as an indicator of pain relief, and pooled results showed the effective rate of pain relief was significantly correlated with acupuncture combined with Chinese medicine, and the heterogeneity was low (MD, 1.22 points; 95% CI, 1.12 to 1.32;*Ⅰ*^2^ = 13%).

**Figure 5.**
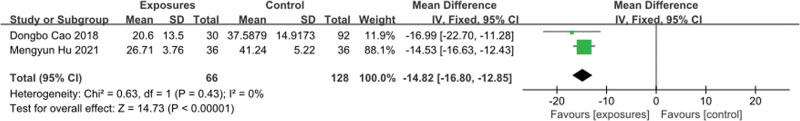
Onset time of analgesia forest map. CI = confidence interval, SD = standard deviation.

**Figure 6.**
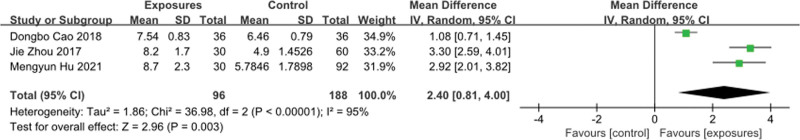
Duration of analgesia forest map. CI = confidence interval, SD = standard deviation.

Two articles^[14,22]^ reported onset time of analgesia measured in minutes(Fig. 5), so MD was employed as the statistical measure for efficacy analysis, pooled results showed significant reduction in the onset time of analgesia was associated with acupuncture combined with Chinese medicine, and the heterogeneity was low (MD, −14.82 points; 95% CI, −16.80 to −12.82;*Ⅰ*^2^ = 0%). Three articles^[14,20,22]^ reported the duration of analgesia(Fig. 6), and the time unit of the 2 articles was hours, so MD was used as the efficacy analysis statistic, pooled results showed that significant reduction in the duration of analgesia was associated with acupuncture combined with TCM, and the heterogeneity was high (MD, 2.40 points; 95% CI, 2.01–3.82;*Ⅰ*^2^ = 95%).

**Figure 7.**
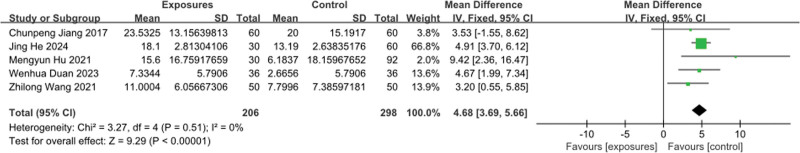
KPS score forest map. CI = confidence interval, KPS = Karnofsky performance status, SD = standard deviation.

With regard to patient quality of life, 5 studies^[13–15,17,18]^ both used Karnofsky performance status(KPS) score(Fig. 7), so MD was used as the efficacy analysis statistic, and pooled results showed the association between life quality and acupuncture combined with Chinese medicine rather than between life quality and other therapies with low homogeneity (MD, 4.68 points; 95% CI, 3.69–5.66;*Ⅰ*^2^ = 0%).

### 3.4. Safety of acupuncture combined with Chinese medicine and other therapies

Minor adverse events were reported in the studies, which did not necessitate medical assessment or intervention. No severe adverse reactions associated with acupuncture were documented. Slight gastrointestinal adverse events were reported in 6 RCTs. One RCT did not report adverse events. One RCT used EORTC QLQ-C30 scale to report slight adverse events. Two RCTs reported slight adverse events based on a reduction in the number of outbreaks of pain. Regarding safety analysis, all 8 RCTs analyzed safety before and after treatment, and only one of them reported abnormal liver function.

### 3.5. Sensitivity analysis

Sensitivity analysis was performed by eliminating each index successively. The findings indicated that after excluding each study individually, the effect size of the KPS score, the total effective rate, and the onset time of analgesia was minimally affected. The sensitivity of the results of the meta-analysis was low, and the conclusions were stable and reliable. However, after excluding study of Jing He, the meta-analysis heterogeneity of numerical descriptor scale scores in acupuncture combined with chinese medication compared with other therapies was reduced (MD, −0.93 points; 95% CI, −1.28–−0.58;*Ⅰ*^2^ = 73%); and after excluding study of Dongbo Cao, the meta-analysis heterogeneity of analgesia duration time in acupuncture combined with chinese medication compared with other therapies was reduced (MD, 3.15 points; 95% CI, 2.59–3.71;*Ⅰ*^2^ = 0%).

## 4. Discussion

A meta-analysis of 10 RCTs involving 884 patients revealed a link between acupuncture in conjunction with Chinese medicine and a more significant decrease in pain intensity, supported by moderate certainty. Additionally, this combination therapy might lead to decreased opioid consumption when used alongside analgesics.^[23–26]^ Few adverse events related to acupuncture or Chinese medicine were documented,^[27–30]^ but there is a lack of comprehensive data on the occurrence of adverse events when acupuncture is combined with TCM. This study presents a recent analysis of the existing evidence on the use of acupuncture in conjunction with Chinese medicine for managing cancer pain and highlights areas requiring further research.

Previous systematic reviews and meta-analyses only evaluated acupuncture or TCM for pain management in patients with cancer pain.^[25,31]^ However, the meta-analysis found that acupuncture combined with Chinese medicine to be associated with greater pain reduction with the simple acupuncture or other therapies, which differs from the findings of previous reviews.

The analysis reveals a consistent trend across multiple studies: acupuncture, when used in conjunction with Chinese medicine, demonstrates a marked ability to alleviate cancer pain. However, when combining the effect sizes results of each study, we found that there was high heterogeneity in pain scores. Through sensitivity analysis, we find that the reason for the high heterogeneity was that the pain score of 1 article was controlled in the range of moderate to severe pain at baseline. There was a high heterogeneity in the duration of analgesia, which was concluded by sensitivity analysis that the reason for the high heterogeneity was that there was no tertiary analgesic method for the intervention in 1 article. Variability in findings indicates that the effectiveness of combining acupuncture with Chinese medicine for cancer pain may vary, making it potentially unsuitable as a standalone treatment. However, the benefits of acupuncture and TCM are not limited to pain relief. They may also strengthen the patients’ immune systems and improve their quality of life. KPS score combined effect size results and safety analysis reveals patients often report improved quality of life, including better sleep, appetite, and emotional well-being. These outcomes are crucial in the management of cancer, where a comprehensive approach to care is essential for optimizing patient outcomes.^[32–35]^ In conclusion, the findings of this systematic review and meta-analysis strongly suggest that acupuncture combined with Chinese medicine offers a viable and effective treatment option for cancer pain. This approach not only provides symptom relief but also contributes to improved overall quality of life for patients. As such, these traditional therapies deserve further exploration and integration into mainstream cancer care protocols. Meanwhile, this study demonstrates that acupuncture therapy is based on a-shi points^[13,15,16,18,19]^ and point combination of the corresponding xi-points, yuan-points, and back-shu points are selected associated with the location of pain and the lesion of zang-fu organs.^[16,18]^ The main method of TCM is external application of ointment,^[13–16,18,20,22]^ and a few are TCM decoction.^[17,19,21]^

The study has several limitations. The 10 included RCTs were all from Chinese authors, resulting in the observation of substantial heterogeneity, and a sensitivity analysis was attempted, which showed reduced heterogeneity in manual pain degree scores and duration of analgesia. The complexity of cancer pain is influenced by various factors such as the type of pain, cancer treatment (e.g., surgery, chemotherapy, hormone therapy), and the phase of care (e.g., active treatment, survivorship, palliative care). The variability in estimates is likely due to factors like acupuncture techniques and prescriptions from traditional Chinese medicine. Further research is necessary to comprehensively evaluate the impact of these factors on the diversity observed.In all the studies, the blinding method was not stated, because it is difficult to blind the treatment of TCM, but did acupuncture achieve blinding by sham acupuncture to ensure that the risk of bias was reduced^[36]^? In the future, the corresponding blinding method should be explored to reduce the risk of bias when using TCM therapy in research.

## 5. Conclusion

The systematic review and meta-analysis conducted in this study yield several key findings regarding the curative effect of acupuncture combined with Chinese medicine on cancer pain.

A significant reduction in cancer pain was observed in patients who received acupuncture in combination with Chinese medicine, compared to those who received conventional treatment alone. However, in the analgesic effect, the differences between the studies were obvious, so the meta-analysis showed that the pain scores of the combined treatment group were not statistically significant. Furthermore, the analysis reveals that the combination therapy is associated with fewer side effects and better quality of life for cancer patients. This is a crucial observation, as cancer pain management often involves a delicate balance between pain relief and adverse effects from medications. The integration of acupuncture and Chinese medicine appears to offer a more holistic and tolerable approach to pain management. The acupuncture therapy is based on a-shi points and the main method of TCM is external application of ointment.A major limitation of this study is that all the RCTs were conducted by Chinese authors. Cultural differences may limit the applicability of these findings to patients in other countries.

## Acknowledgements

All the authors of this article would like to express their gratitude to all the members of Acupuncture and Moxibustion Consulting Room No. 10 in the Second Affiliated Hospital of Heilongjiang University of Traditional Chinese Medicine.

## Author contributions

**Conceptualization:** Guocheng Xue.

**Data curation:** Guocheng Xue, Siqi Zhang, Afeng Miao, Yubo Teng, Hao Ming.

**Formal analysis:** Guocheng Xue.

**Investigation:** Guocheng Xue.

**Methodology:** Guocheng Xue.

**Project administration:** Guocheng Xue.

**Resources:** Miao Zhang.

**Software:** Guocheng Xue.

**Supervision:** Miao Zhang.

**Validation:** Guocheng Xue.

**Visualization:** Guocheng Xue.

**Writing – original draft:** Guocheng Xue.

**Writing – review & editing:** Guocheng Xue, Miao Zhang.
